# Effects of an anti-IGF-1 receptor monoclonal antibody on laminitis induced by prolonged hyperinsulinaemia in Standardbred horses

**DOI:** 10.1371/journal.pone.0239261

**Published:** 2020-09-29

**Authors:** Samira Rahnama, Niveditha Vathsangam, Robert Spence, Carlos E. Medina-Torres, Christopher C. Pollitt, Melody A. de Laat, Simon R. Bailey, Martin N. Sillence

**Affiliations:** 1 Biology and Environmental Sciences School, Queensland University of Technology, Brisbane, Queensland, Australia; 2 Faculty of Veterinary and Agricultural Sciences, The University of Melbourne, Melbourne, Victoria, Australia; 3 School of Veterinary Science, The University of Queensland, Gatton, Queensland, Australia; University College Dublin, School of Veterinary Medicine, IRELAND

## Abstract

Currently, there are no registered veterinary drugs for the treatment of endocrinopathic equine laminitis, and although this form of the disease is known to be caused by prolonged hyperinsulinaemia, the mechanism of insulin toxicity is unclear. One possibility is that high concentrations of insulin activate IGF-1 receptors (IGF-1R) in lamellar tissue, leading to uncontrolled cell proliferation and epidermal lamellar dysregulation. An equinized version of a human anti-IGF-1R therapeutic monoclonal antibody (mAb11) was generated to test this theory, using a modification of the prolonged euglycaemic-hyperinsulinaemic clamp technique. Healthy Standardbred horses were infused for 48 h with 0.9% saline (negative-control, n = 6), a combination of insulin (4.5 mIU/kgBW/min) and a variable infusion of 50% glucose to maintain euglycaemia (positive-control, n = 6), or insulin and glucose, preceded by a low dose of mAb11 (20 mg), designed to treat one foot only and delivered by retrograde infusion into one forelimb (mAb-treated, n = 7). Maximum insulin concentrations were 502 ± 54.4 and 435 ± 30.4 μIU/mL in the positive-control and mAb11-treated groups, respectively (P = 0.33). While the control group remained healthy, all the insulin-treated horses developed laminitis within 30 h, as judged by clinical examination, foot radiographs and histological analysis. Some effects of insulin were not attenuated by the antibody, however, relative to the positive-control group, horses treated with mAb11 showed less sinking of the distal phalanx (P < 0.05) and milder histological changes, with markedly less elongation at the tips of the secondary epidermal lamellae (P < 0.05). These differences were apparent in both front feet and were statistically significant when the values for both feet were combined. The results confirm that IGF-1R may have a role in insulin-induced laminitis and suggest that mAb11 warrants further research as a potential agent to prevent or treat the disease.

## Introduction

Because there is no convenient cure for endocrinopathic equine laminitis, a common disease of the horse’s foot, the condition has been known to claim the lives (through euthanasia) of up to 33% of affected animals within 12 months of diagnosis [1], and to recur in 34% of individuals within two years [2].

The development of effective treatments for this form of laminitis has been hampered by an incomplete understanding of the pathophysiology of the disease. It has been clearly established that insulin dysregulation is a primary underlying factor, as hyperinsulinaemia is commonly observed in naturally-occurring cases [3], and in a dietary induction model, where the risk and speed of onset of the disease were clearly associated with postprandial insulin concentrations [4]. Further, laminitis can be induced in healthy ponies and horses through the prolonged infusion of insulin and glucose [5, 6].

However, the few insulin receptors in the lamellar tissue [7] are confined to the microvasculature [8], suggesting an indirect mechanism of action, whereby the pathological effects of insulin are mediated by the overstimulation of IGF-1 receptors (IGF-1R) in the epidermal lamellar tissue [9]. There is some conflicting evidence in this regard, as insulin appears to have a relatively low affinity for binding to IGF-1R in equine lamellae [7]. Conversely, the hypothesis is supported by the observation that IGF-1R gene expression is downregulated during the insulin-induction model *in vivo*, in the absence of elevated plasma IGF-1 concentrations [9]. In addition, insulin can stimulate cell proliferation in isolated lamellar cells *in vitro*, and that this effect can be blocked using a selective anti-human IGF-1R monoclonal antibody (mAb) [10].

To explore the mechanism of insulin toxicity *in vivo*, and the potential use of an anti-IGF-1R mAb as a therapeutic agent for laminitis, we developed an equinized mAb based on a human antibody, with the intention of avoiding an immune response in its recipients. The difference between the human and equine versions of the IGF-1RmAb is based on the Fc portions of the mAb, along with a few functional amino acid changes to the variable regions of the antibody in the equine version alone.

Using this antibody, we set out to test the hypothesis that the treatment can prevent or attenuate the effects of insulin infusion on the feet of live horses. A secondary aim of the study was to improve the prolonged euglycaemic-hyperinsulinaemic clamp (p-EHC) technique, which has previously employed a supra-physiological concentration of insulin to induce laminitis. Our objective was to induce clear signs of laminitis within 48 h, while keeping insulin concentrations within the high physiological range, seen naturally in insulin-dysregulated horses after feeding.

## Materials and methods

### Animal selection, characteristics and management

This project was approved by the Animal Ethics Committees of the University of Queensland (Approval # QUT/SVS/457/18) and Queensland University of Technology (Approval # 1800001230), and was conducted in strict accordance with the approval.

The study design was a randomised, unbalanced, controlled trial, using 19 Standardbred horses purchased from a local dealer in South-East Queensland. The herd included 11 mares and 8 geldings, with a mean (±SE) BW of 445 ± 8.4 kg, age 5.6 ± 0.7 years, height 1589 ± 12 mm; and a median (range) BCS of 4.5/9 (3.5–6), and a cresty neck score of 0/5 (0–1.5).

Three horses of similar body weight (BW) were transported to the experimental horse unit each week, except in week 6 when four horses were delivered. The horses were examined by a veterinarian for general health status, as well as undergoing a thorough lameness examination using the modified Obel method developed by Meier et al. [11]. The clinical examination included: BW, body condition score (BCS), cresty neck score (CNS), demeanor, thoracic and abdominal auscultation, heart rate, respiratory rate, temperature, forelimb digital pulse palpation, capillary refill time, mucous membrane colour, skin turgor, lymph node palpation, and visual inspection for any abnormalities.

After being examined, measured for height using a tape, and weighed on electronic scales, the horses were placed in adjacent individual stables for 24 h, with free access to water, lucerne (alfalfa) hay and lucerne chaff.

The next day, the horses were allocated at random to one of three groups. Randomization was achieved by pre-determining the order of treatment allocation, then assigning each horse to one of three groups according to this list and their order of presentation (ID number). The experimental groups were: negative-control (0.9% saline infusion only), positive-control (infused with insulin and glucose), and mAB11-treated group (pre-treated with mAb11, then infused with insulin and glucose). The experiment was replicated each week for 6 weeks, with an additional horse tested in week 6 allocated to the mAb11-treated group.

### Experimental compounds

The negative-control horses received 0.9% saline (Baxter Healthcare, NSW, Australia). Positive-control horses received infusions of recombinant human insulin (Humulin-R™; Eli-Lilly Australia, West Ryde, NSW, Australia) and a 50% solution of glucose (Baxter Healthcare, NSW, Australia). The treated group received insulin, glucose and a fully equinized anti-IGF-1R mAb (mAb11). The mAb was developed by our group and validated for receptor binding affinity and specificity in our laboratories using a range of techniques including radioligand binding studies with fresh equine liver and lamellar tissue, flow cytometry studies using cloned equine IGF-1R, receptor phosphorylation studies, and cell proliferation studies [12, 13].

### Experimental procedures

At the start of each infusion phase, the horses underwent a second clinical examination to determine there were no signs of illness, followed by a lameness examination and the application of a radio-opaque marker (barium) to the dorsal midline of each front hoof. The hooves were radiographed and fourteen-gauge extended-use intravenous (i.v.) catheters (Mila International, Florence, KY, USA) were aseptically placed in the left and right jugular veins of each horse, for the administration of the infusions (right) and blood sampling (left). The horses were placed in individual stocks, where they remained for most of the time during the 48-h infusion period. During their time in the stocks, the horses had continuous access to fresh water, hay and chaff.

Before the systemic infusions commenced, one of the two horses to be infused with insulin was also given a dose of 20 mg mAb11 dissolved in 10 mL saline. The mAb was administered by retrograde infusion into the palmar digital vein of the left front leg, at the level of the metacarpophalangeal (fetlock) joint. The dose was based on the typical amount of anti-IGF-1R mAb delivered to human cancer patients in clinical trials [14, 15], and was designed to treat one hoof with an approximate weight of vascularised tissue of 1 kg. The estimate of hoof weight was based on abattoir samples. The horse from the positive-control (insulin-only) group received 10 mL of saline alone. To perform the infusions safely, each horse was first sedated with i.v. xylazine (0.4 mg/kg BW, Troy Laboratories, Sydney, NSW, Australia) and given a perineural anaesthetic (lignocaine; 10 to 20 mL, Mavlab, Logan City, Qld., Australia) by subcutaneous injection over the palmar digital nerve. A tourniquet was then fitted to the leg at the level of the proximal sesamoid bones prior to the retrograde infusion, and left in place for 20 min following the injection, to enable the mAb to perfuse the dermal lamellar tissue. The 10 mL retrograde infusion was delivered through a winged infusion set (butterfly needle) over 5-min period.

The insulin and glucose infusions used in this study were similar to those administered to Standardbred horses in an earlier study [6] to perform a euglycaemic-hyperinsulinaemic clamp (EHC), with a slight modification. As the EHC has been criticised for raising insulin concentrations beyond the physiological range, the dose of insulin delivered was reduced by 25%, and the infusion period was limited to 48 h.

Thus, of the 19 horses use in the study, 13 received a bolus of insulin (45 mIU/kgBW), followed by a constant infusion of insulin (4.5 mIU/kgBW/min), with a variable infusion of 50% glucose, commencing at 10 μmol/kgBW/min. Negative-control horses received an intravenous infusion of 0.9% saline, at a volume and rate equivalent to the total volume of liquid delivered to the EHC horses.

### Blood samples and maintenance of euglycaemia

Blood samples were taken from all horses, commencing 1 h before and at regular intervals throughout the experiment. A pre-infusion sample (10 mL) was collected to confirm that serum biochemistry and whole-blood haematology parameters were normal. Additional samples (5 mL) were collected at times -30 min, 0 min, then after 1, 2, 3, 4, 5, 6, 7, 8, 10, 12, 18, 24, 30, 36, 42 and 48 h relative to the start of the infusions, for serum insulin analysis. Finally, 1 mL samples were collected every 5 min, commencing 15 min before the infusion and continuing for the next 3 h, for the immediate measurement of blood glucose in the groups that received an insulin infusion. Based on the result of each glucose measurement, the glucose infusion rate was adjusted to maintain a concentration of 5 mM (+/- 1 mM). Once the glucose level had stabilised, blood glucose was measured every 30 min until the end of the experiment. Blood glucose was measured in the negative-control horses every 6 h.

Blood samples for serum analysis were collected in plain vacutainer tubes (Becton Dickinson, Sydney, NSW, Australia). After standing for 30 min at room temperature, the samples were centrifuged at 3000 x g for 30 min. Aliquots of serum (1 mL) were transferred to eppendorf tubes and stored at -20°C until further analysis, which was conducted within three weeks. Plasma samples were collected into chilled, EDTA-coated tubes (Becton Dickinson, Sydney, NSW, Australia), which were stored at 4°C before being processed as described above.

Blood glucose concentrations were measured on fresh whole blood a using a hand-held glucometer validated previously for use in the horse [4] (Accucheck Go, Castle Hill, NSW, Australia). Serum insulin and plasma ACTH were measured by a commercial laboratory (Vetpath Laboratory Services, Perth, WA, Australia) using chemiluminescent assays (Immulite XPi, Siemens Healthcare Diagnostics, Bayswater, Vic, Australia) validated previously for use in the horse [16]. In addition, routine haematological and biochemical measurements were made by another commercial diagnostics laboratory (QML, Brisbane, Qld., Australia).

### Animal monitoring

In addition to the lameness and clinical examinations conducted prior to the infusion, the horses were examined every 6 h during the infusion period for any indications of pain, including changes in demeanour, sweating, posture, respiratory rate, heart rate and rectal temperature. The horses were also disconnected from the infusion apparatus and subjected to a lameness exam every 6 h until signs of pain and/or lameness were detected. Once any pain or lameness was observed, the horses were given pain relief (phenylbutazone 2.2 mg/kgBW, Troy Laboratories, Sydney, NSW, Australia). Clinical examinations were continued every 6 h until the end of the experiment to monitor the efficacy of the pain medication, and if signs of pain persisted, a further dose of phenylbutazone administered. No further lameness examinations were performed, however, as the medication was expected to mask these signs.

### Radiographic assessment

Radiographic images (lateromedial projection) of both forelimb hooves were taken before the study and just prior to euthanasia. A thin line of barium sulphate (incorporated into polyurethane resin) was applied to the dorsal hoof wall (midline) and the dorsal sole. The polymerised resin/barium sulphate mixture adhered to the hoof wall for the duration of the experiment and being permanent, optimised the accuracy of radiographic measurements. To identify any signs of rotation or sinking of the pedal bone, three measurements were made of the distance between the radiopaque marker on the hoof wall (HW) and the parietal surface of the distal phalanx (DP) at the proximal, medial and distal regions. A fourth measurement was made from the tip of the DP to the radiopaque marker on the sole of the foot (S1 Fig). Finally, an attempt was made to measure the founder distance (between the top of the barium line on the dorsal hoof wall and the proximal limit of the extensor process of the DP), but this measurement, which averaged only 2 mm in all feet before treatment, yielded high coefficient of variation (93%), and was considered too inaccurate to detect the subtle changes caused by insulin.

The measurements were performed by two investigators (CCP and RS) who were blinded to the treatment of each horse, using Sante DICOM Viewer Pro V11.1 software (Santesoft, Nicosia, Cyprus).

### Tissue collection

At the end of the experiment, the horses were sedated with xylazine (1 mg/kgBW; Troy Laboratories, Sydney, NSW, Australia) prior to euthanasia by an injection of sodium pentobarbital (150 mg/kg BW, i.v., Lethabarb, Virbac, Sydney, NSW, Australia). Following euthanasia, the forelimbs were collected immediately by disarticulation at the pastern. Sagittal sections were cut using a bandsaw and then trimmed using a scalpel. Lamellar sections (5 mm × 5 mm) were fixed using 10% neutral buffered formalin (Sigma-Aldrich, Sydney, NSW, Australia), left overnight in formalin, then transferred to 70% ethanol and stored for further investigation.

### Histology and histomorphometry

The stored explants were embedded in paraffin and cut into 5–7 μm sections, before being stained with Periodic acid-Schiff (PAS) using an Autostainer (Leica Autostainer XL, Leica, Nussloch, Germany). Sections were imaged with a 3D Histech scan II fluorescence/Brightfield slide scanner (3DHistech, Budapest, Hungary) with a total magnification of 70x and 0.14 μm resolution. The images were then analysed using CaseViewer Software (3DHISTECH, Budapest, Hungary).

The distal section images were examined by two blinded veterinary researchers with significant experience of lamellar histopathology (CP and SB), who provided a subjective qualitative assessment of the images and a laminitis severity score based on a published method developed to assess laminitis induced by oligofructose overload [17, 18]. There was an acceptable level of agreement between these observers, with a Spearman Rank correlation co-efficient of 0.83.

Histomorphometric analysis was performed blind by two investigators (SR and RS) according to a protocol published previously [18]. This included measuring the lengths of primary epidermal lamellae (PEL) and secondary epidermal lamellae (SEL) at the abaxial (SELB) and axial (SELA) end of the PEL, and the widths of SEL on either side of the PEL, with the basement membrane oriented to the right and the upper and lower sides of the PEL being designated accordingly. Eight consecutive PELs were selected from each section and separate measurements were performed for each front foot of each horse as described in S2 Fig.

### Statistical analyses

Parametric data were subjected to the Shapiro-Wilk test for normality. Radiographic measurements of the change in the HW-to-DP distance, and the DP-to-sole distance over the course of 48 h, revealed considerable variation between horses and between feet, providing limited power to detect any significant changes. However, given there was a numerical increase for the insulin-treated group, and a numerical decrease for the mAb11 group (relative to insulin only) in the DP-sole distance and in the HW-DP distance at all locations measured, the results for the left and right feet were combined, in an attempt to lessen the variation and determine if the specific effects of treatment became clearer.

For histology data, epidermal lamellar measurements for both fore-feet were also averaged and compared using one-way analysis of variance (ANOVA). The Holm-Sidak method was used to compare the positive-control (insulin only) group with the negative control (untreated) and mAb-treated groups. For histological scores (assigned on a scale of 1 to 3), the values for both feet reported by both assessors were averaged and compared using ANOVA on ranks, followed by Dunn’s method. Insulin concentrations were plotted against time and the area under the curve was calculated for each horse. As these data did not conform to a normal distribution, they were also analysed using ANOVA on Ranks and Dunn’s method. Ordinal data, including the initial time to develop laminitis were averaged (median) and compared between inulin and insulin- mAb11 groups using a Mann-Whitney Rank Sum Test. All data are presented as mean ± SE, or median (range). Statistical significance was set at P < 0.05. All analyses were conducted using SigmaPlot 13 software (Systat Software, San Jose, CA).

## Results

### Insulin concentrations

**Fig 1:**
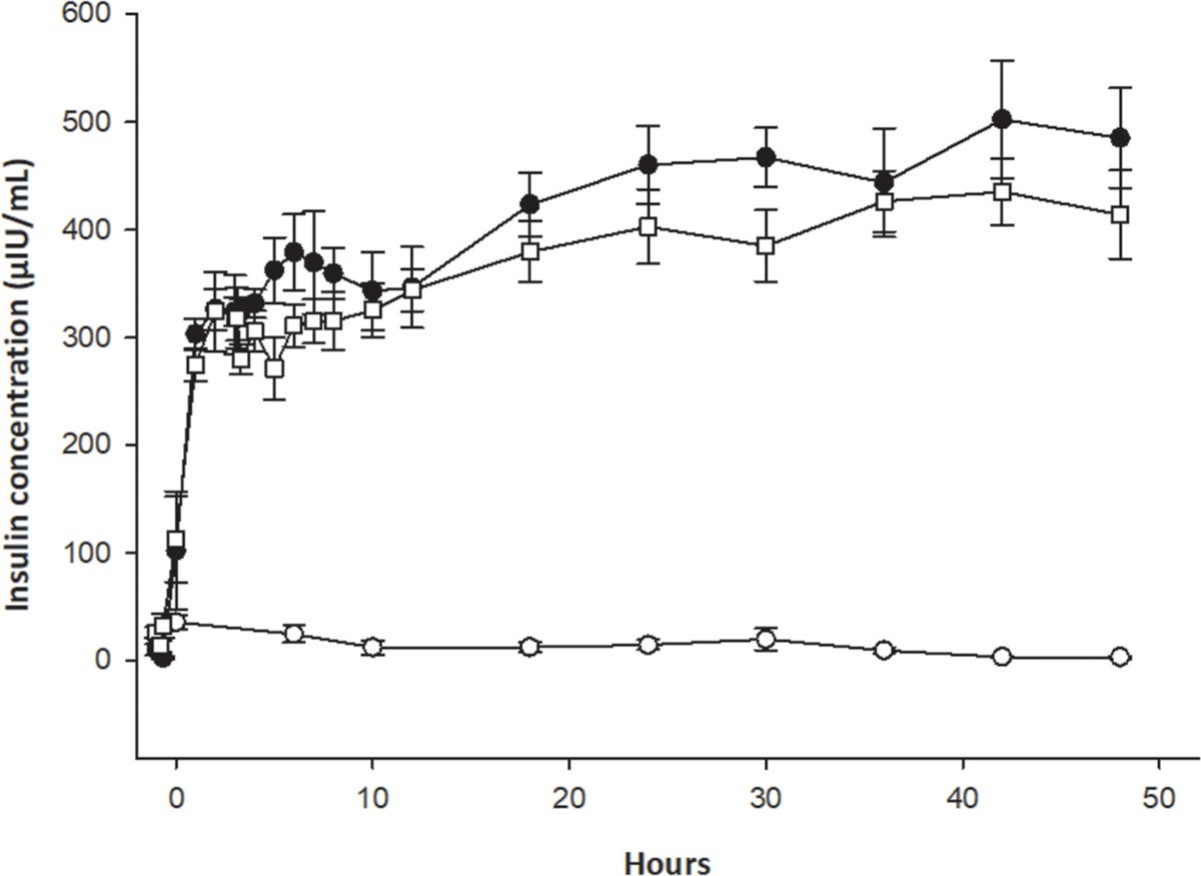
Serum insulin concentrations (mean + SE) in three groups of horses given a constant infusion of saline (○), insulin (●), or insulin plus a monoclonal anti-IGF-1R antibody (□). According to the area under the curve, insulin concentrations were lower in the negative-control group (P < 0.01), but not different between the positive-control and antibody-treated groups (P = 0.99).

The median (IQR) insulin concentration in samples from all horses taken between 1 h and 30 min before the infusions commenced was 8.3 ± 27.5 μIU/mL. Immediately before the infusions commenced (time 0), the median insulin concentrations in the negative control (44 μIU/mL), positive control (36 μIU/mL) and mAb11-treated groups (31 μIU/mL) were slightly elevated, with no difference between these groups. From 6 h through to 48 h the insulin concentration remained low and stable in the negative-control group. However, in the horses given an insulin infusion, serum insulin increased rapidly over the first 2 h of the infusion period, followed by a more gradual increase over the next 46 h (Fig 1).

Maximum insulin concentrations were reached after 42 h in the positive-control and mAb11-treated groups, and were of similar magnitude (502 ± 54.4 and 435 ± 30.4 μIU/mL, respectively; P = 0.33). There was also no difference between these groups when the area under the plot of insulin concentration versus time was calculated for each horse (P = 0.99). However, insulin concentrations were markedly lower over the duration of the study in the negative-control group (P < 0.01), as expected (Fig 1).

### Lameness observations

All 13 horses infused with insulin developed clinical signs of lameness within 30 h, varying in severity from a score of 1 to 7 on the “modified Obel” scale. The lowest score on this 12-point scale of indicates an abnormality in only one of five variables measured, which include: a bounding digital pulse, weight shifting, resistance to lifting the front feet, walking in a straight line, and turning in a circle [11]. The median time for the first signs of lameness to appear was 24 h for the positive-control group, and 18 h for the insulin + mAb group, with a range of 12–30 h for both groups (P = 0.534). The median (and range) of severity scores was 4 (2–7) for the positive-control group and 2 (1–4) for the insulin + mAb group (P = 0.14). None of the negative-control horses exhibited any signs of lameness.

### Radiographic analysis

**Fig 2:**
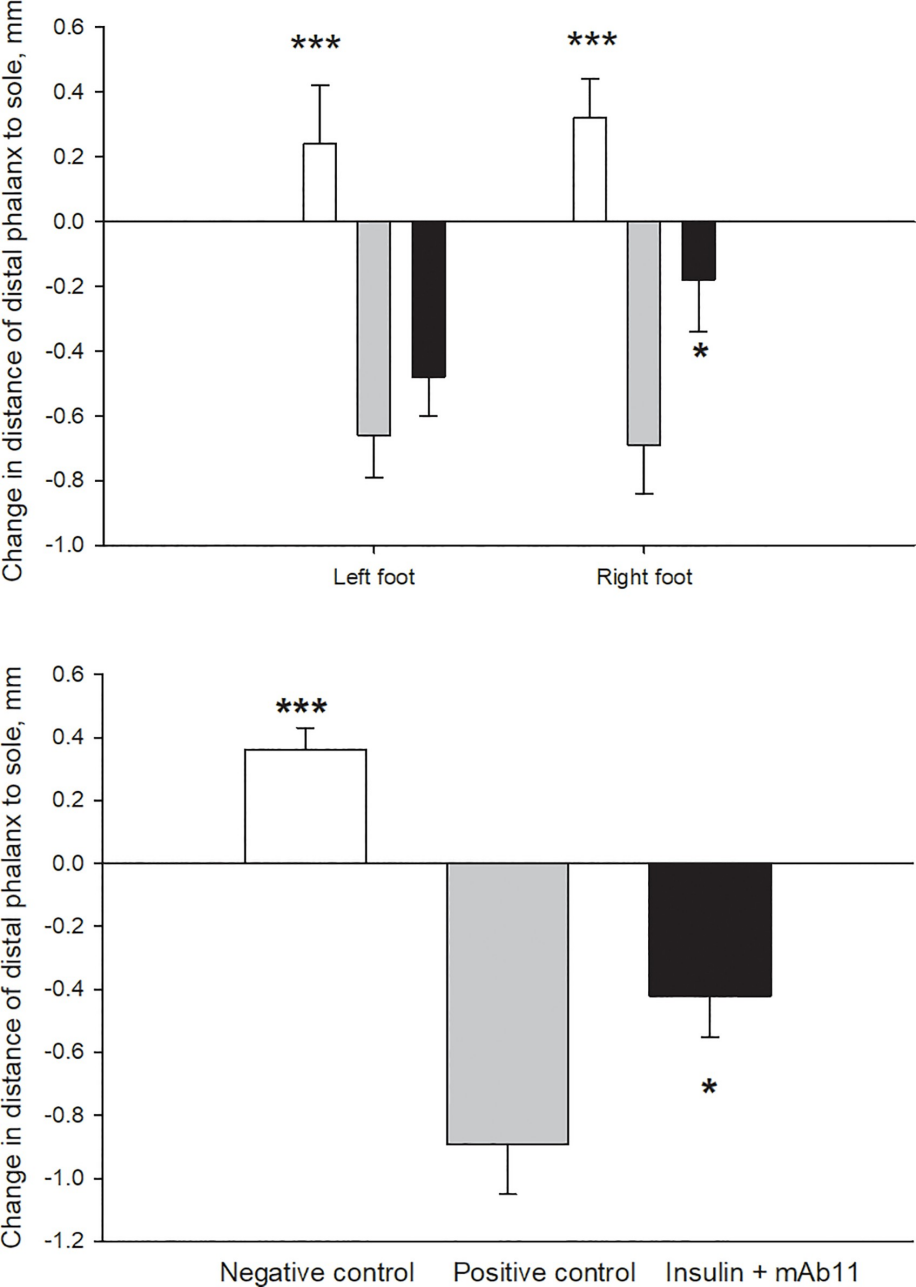
Change in the distance between the tip of the distal phalanx and the sole of the foot, measured using radiography. Values represent individual feet (a) or are averaged between the left and right front feet (b) in three groups of horses treated with: 0.9% saline (negative-control, n = 6, white bars); a combination of insulin and glucose infusion (positive-control, n = 6, grey bars); or a combination of insulin and glucose, plus an anti-IGF-1R monoclonal antibody (mAb11, n = 7, black bars) delivered to the left foot by local infusion. *P < 0.05 and ***P < 0.001, compared with the positive-control group. The negative control group was not significantly different to zero.

**Fig 3:**
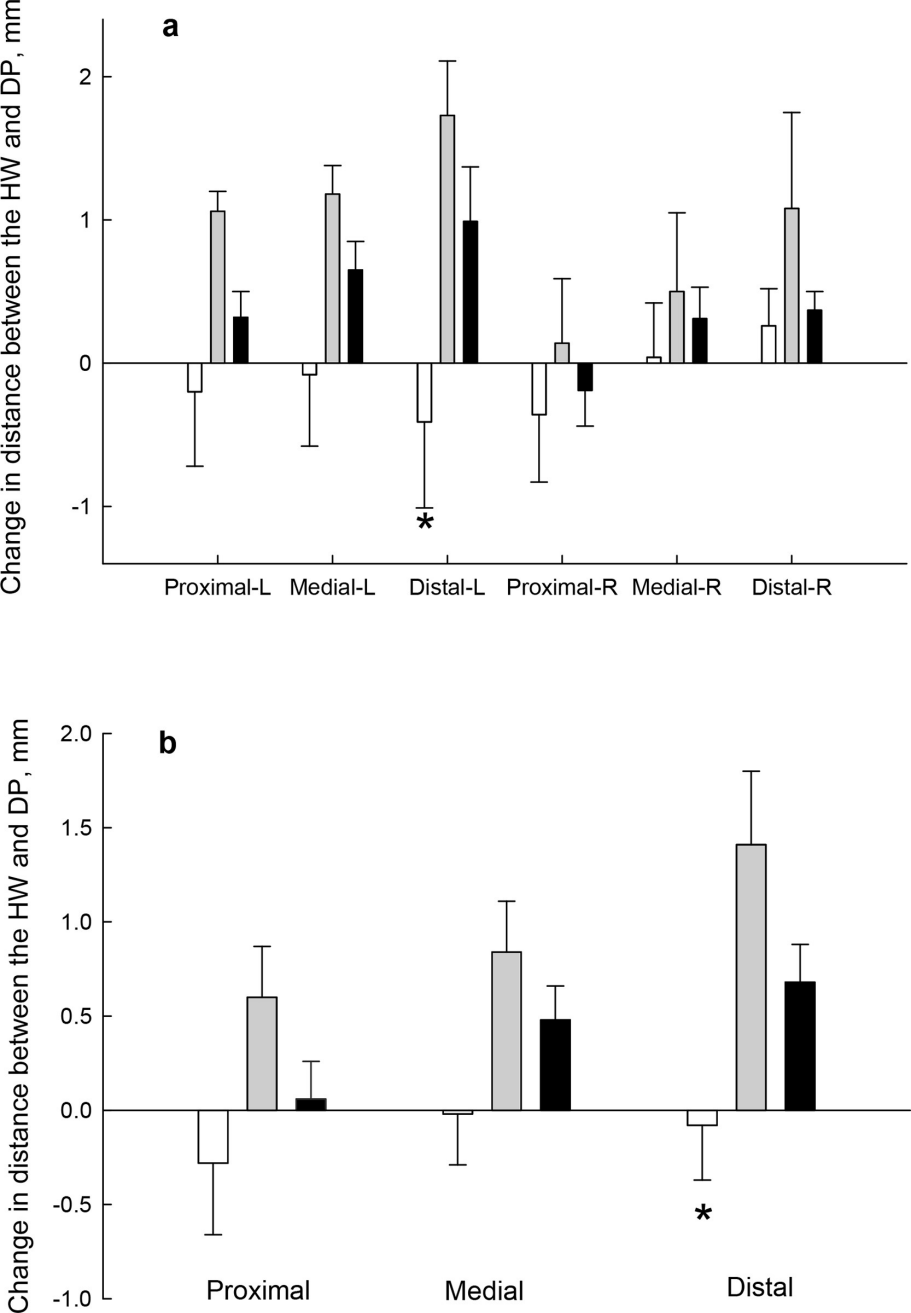
Change in the distance between the hoof wall (HW) and the distal phalanx (DP), measured using radiography. The values represent individual feet (a) or the average for the left and right front foot of each horse (b), belonging to one of three groups treated with: 0.9% saline (negative-control, n = 6, white bars); a combination of insulin and glucose infusion (positive-control, n = 6, grey bars); or a combination of insulin and glucose, plus an anti-IGF-1R monoclonal antibody (mAb11, n = 7, black bars). *P < 0.05 compared with the positive-control group.

Radiographic measurements of the change in the HW-to-DP distance, and the DP-to-sole distance over the course of 48 h, are shown in Figs 2 and 3, respectively. For the combined feet, in the insulin-treated positive-control group, the distance between the DP and the sole was decreased relative to the negative controls (Fig 2B; P < 0.001) and the DP-HW distance at the tip of the DP was increased (Fig 3B; P = 0.018), indicating that sinking or rotation of the DP, relative to the hoof wall had occurred.

Compared with the positive-control group, the change in the DP-sole distance was less in the mAb11-treated horses (P = 0.019), whereas there was no significant change in HW-DP distance (P = 0.079). Further, in the mAb11 group, there was no apparent difference between the mAb11-treated right foot and the untreated left foot, in the degree to which the effects of insulin were attenuated.

### Histopathology

#### Negative-control (saline) group

**Fig 4:**
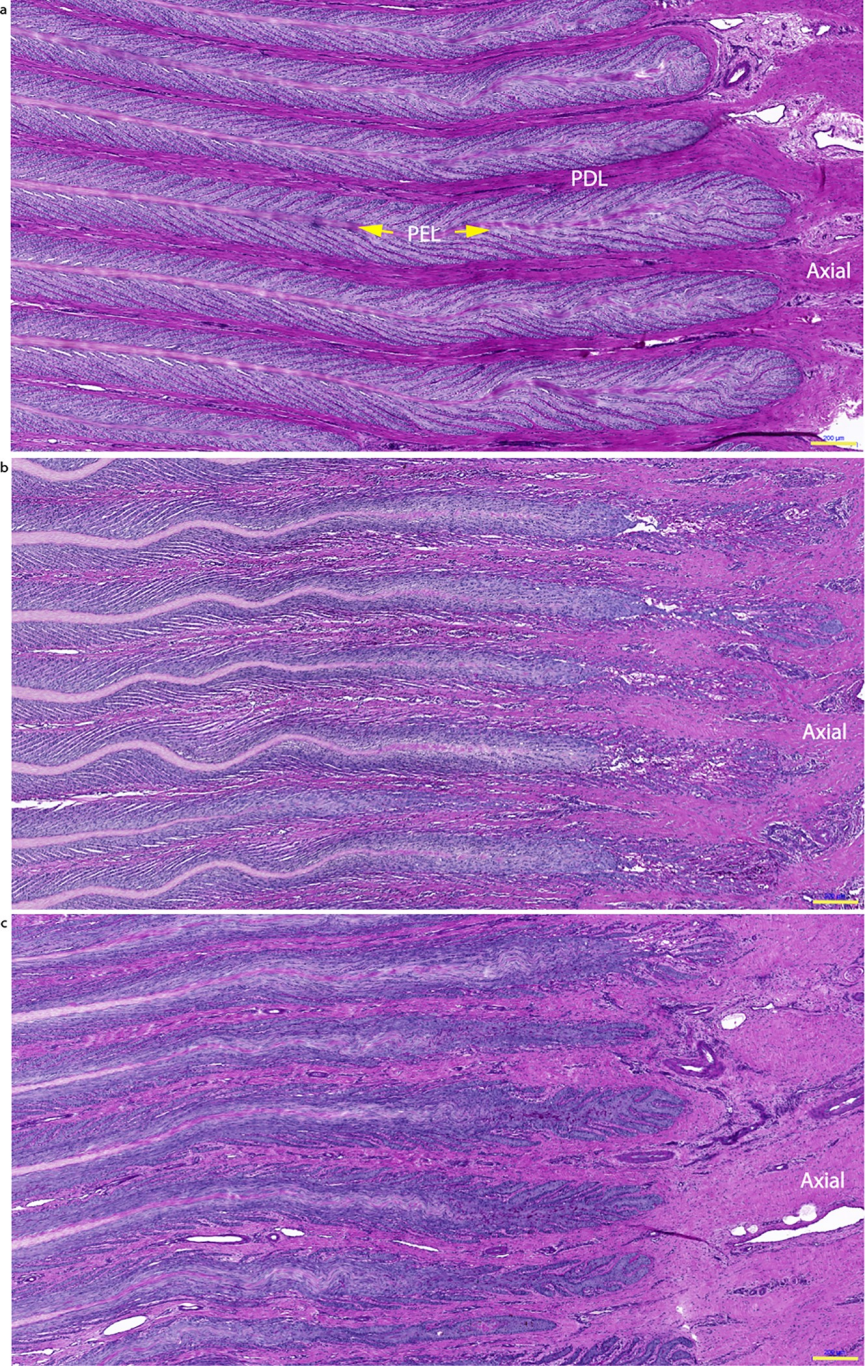
Photomicrographs of hoof lamellar histology, viewed at the transverse distal section of the lamellae. Representative samples are shown from a negative-control horse (a), a positive-control, insulin-treated horse (b) and a horse treated with insulin plus an anti-IGF-1R monoclonal antibody (mAb11) (c). The primary epidermal lamellae (PEL) were normal in control horses, severely elongated and distorted in the positive-control group, and slightly elongated in the insulin + mAb11 group (Scale bar = 200 μm).

Two blinded expert assessors, who performed a qualitative analysis of the lamellar structure in sections from the forelimbs, determined that the tissue was normal in most of the samples from the negative-control horses, with only slight aberrations in some samples (Fig 4A). The median (range) laminitis score assigned to this group and averaged across both feet was 0.13 (0–0.75), on a scale of 0 to 3. Values for individual feet are presented in S1 Table.

#### Positive-control group (insulin-only)

**Fig 5:**
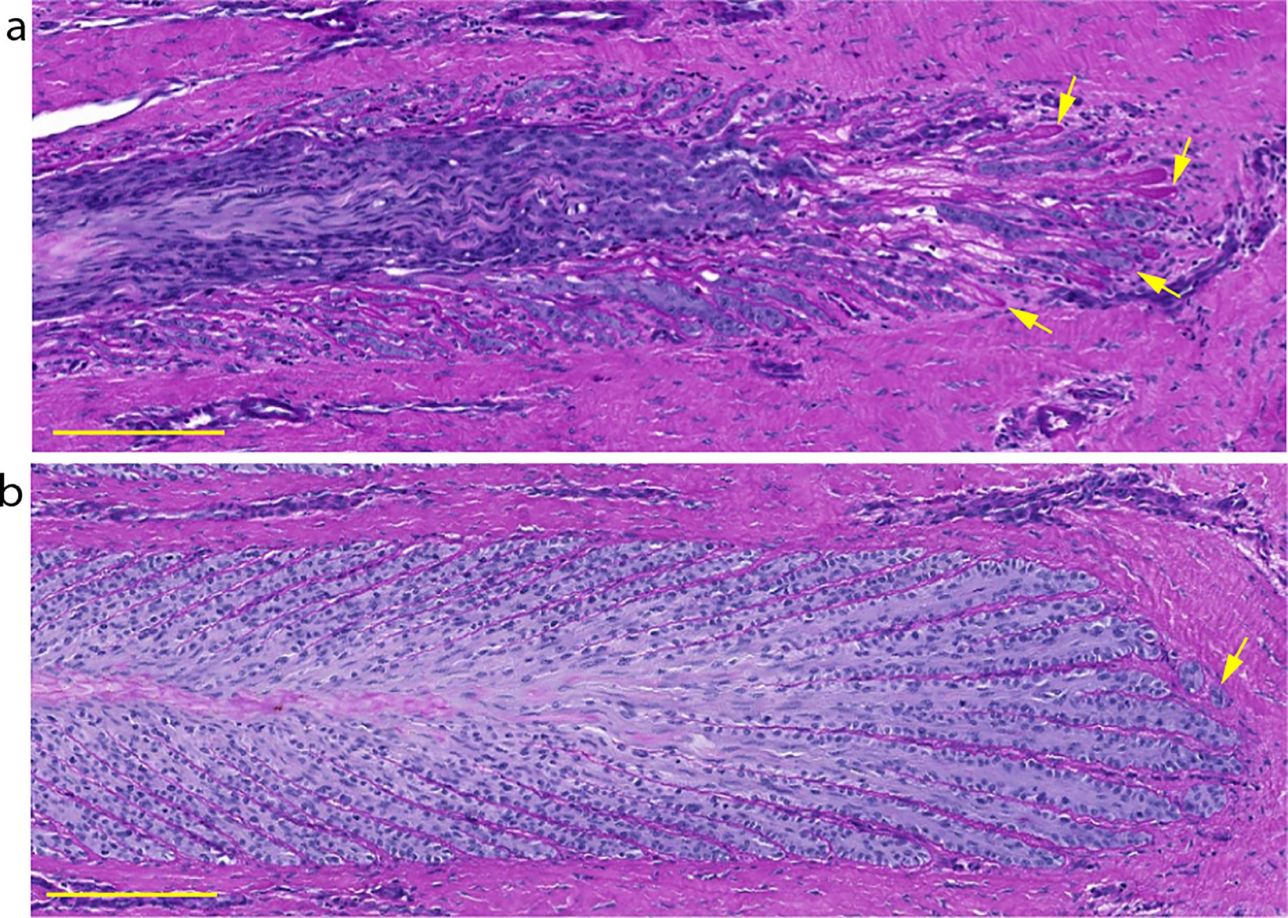
Photomicrographs of hoof lamellar histology, viewed at the transverse distal section of the lamellae. Representative samples are shown from a positive-control, insulin-treated horse (a), and a horse treated with insulin plus an anti-IGF-1R monoclonal antibody (mAb11) (b). In sample ‘a’ the basement membrane is completely separated from the basal cells, whereas in sample ‘b’ the BM is still attached. The arrows show empty basal membrane and isolated EBS cells (Scale bar = 50 μm).

**Fig 6:**
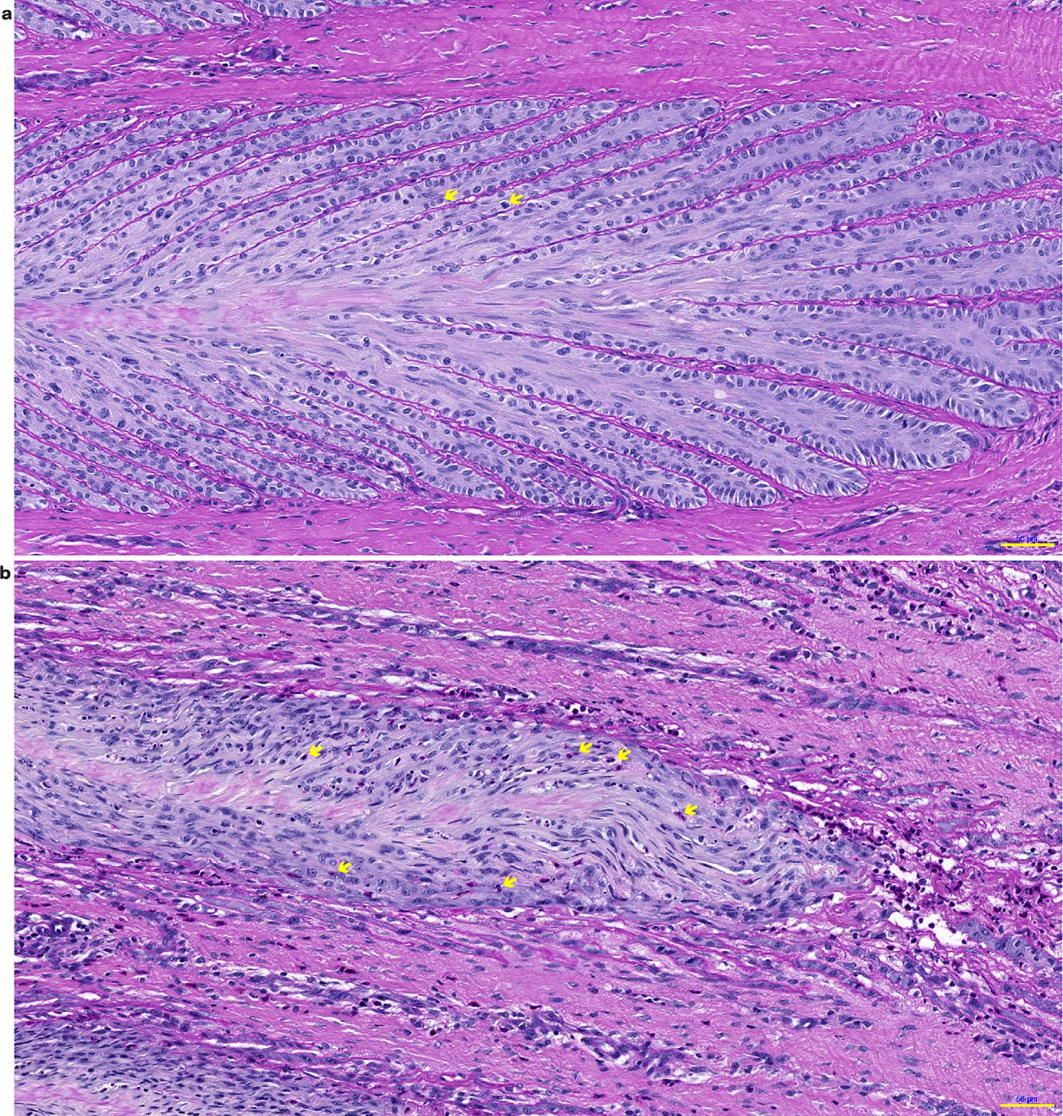
Photomicrographs of hoof lamellar histology, viewed at the transverse distal section of the lamellae. Representative samples are shown from a positive-control, insulin-treated horse (a), and a horse treated with insulin plus an anti-IGF-1R monoclonal antibody (mAb11) (b). Evidence of mitosis can be seen in both samples, with the arrows indicating mitotic figures (Scale bar = 50 μm).

**Fig 7:**
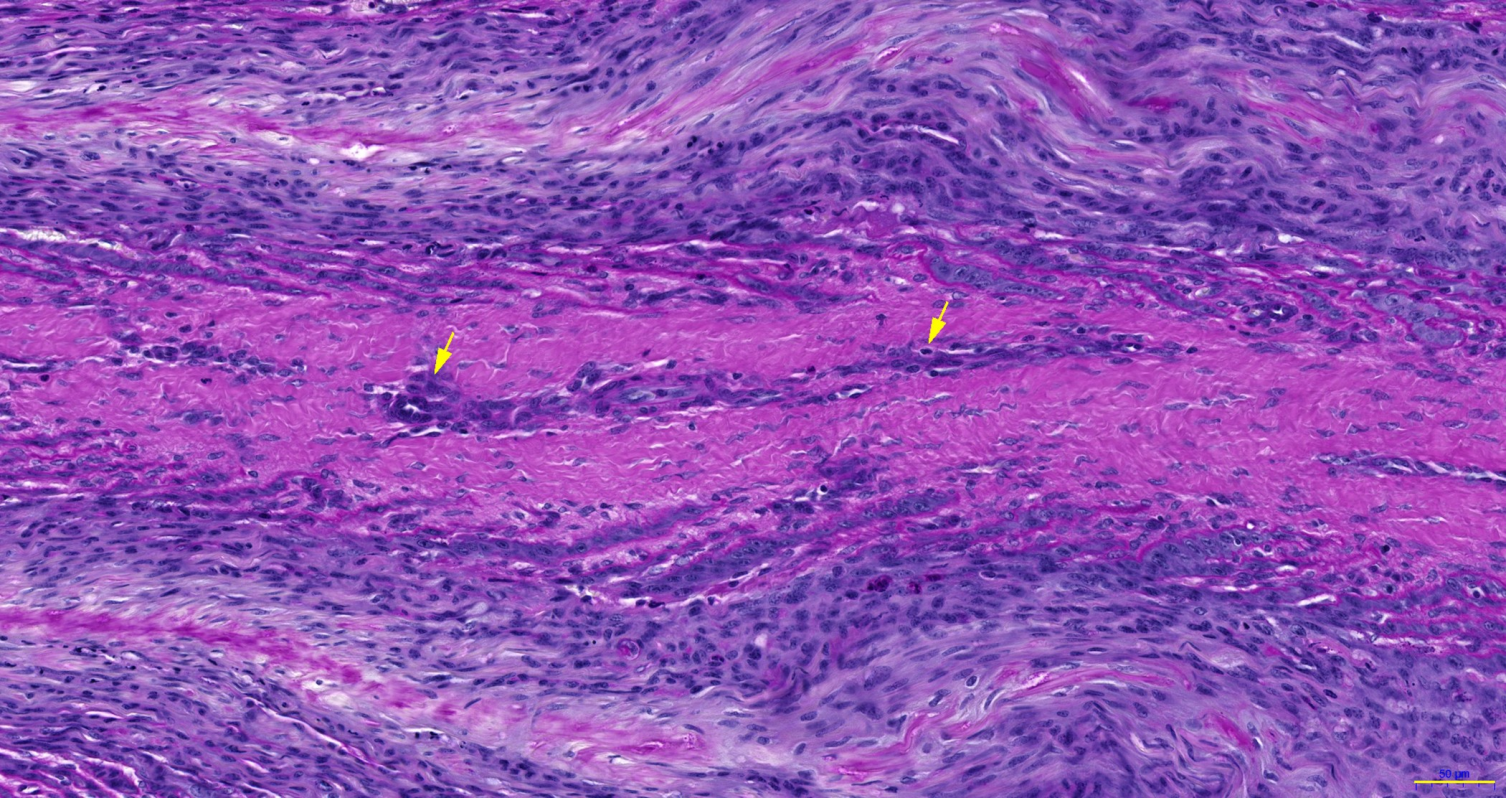
Photomicrograph of hoof lamellar histology, viewed at the transverse distal section of the lamellae. A representative sample is shown from a positive-control, insulin-treated horse. The arrow indicates inflammatory cells, with granules that show strong PAS-positive staining in the primary dermal lamellar vessels (Scale bar = 50 μm).

In the sections from positive-control horses, the PEL were typically elongated axially and distorted (Fig 4B). The SEL were severely elongated, especially at the axial end (tip) of each PEL, and dermo‐epidermal separation was apparent. In the distal region (axial) of the PEL, the basement membrane (BM) was fragmented, detached, and (or) absent at the base of the SEL (Fig 5A). However, in the middle and proximal regions of the PEL, the BM was drawn out into double-layered strands in some samples. Many epidermal cells had nuclei that appeared rounded, compressed and tilted, and many had lost their apical position in the EBC cytoplasm. Mitotic figures and apoptotic EBC were frequently observed in the SEL located at the tips of the PEL, where the SEL were separated (Fig 6A). Inflammatory cells occasionally appeared at the axial end of each PEL and around areas where tissue damage was apparent (Fig 7). The median (range) score for this group of horses was 2.38 (1–3), which was significantly higher than that for the negative-control group (P = 0.001).

### Treatment group (insulin + mAb11)

The histopathology of the horses that received insulin and mAb11 showed many similarities to that of the insulin-only positive-control group, but with some important differences (Fig 4C). In particular, the SEL located at the axial end of each PEL were less elongated, and the basement membrane was still attached to the SEL (Fig 5B). Few samples showed any evidence of mitotic figures, apoptosis, or inflammatory cells. The median score (range) given to this group was 1.75 (0.75–2.5), which was not significantly different to the positive control group.

### Histomorphometry

Detailed results of histomorphometry measurements showing values for both front feet are presented in S2 Table. As there were no significant differences in any variable between samples from the left and right foot, the data for both feet were averaged.

When the positive-control and the negative-control groups were compared, the signs of insulin-induced laminitis were clear and marked, with an increase in the length of the PEL and SEL, and a decrease in the width of the SEL, consistent with stretching of the tissue. However, not all the variables were affected evenly, with changes in total PEL length (TPELL), keratinised PEL length (KPELL), and the length of the SEL at the abaxial end (SELLB), increasing by between 9% and 48%. The width of the SEL at the upper side (SELWU) and lower side (SELWL) of the PEL, was decreased by 48% and 53%, respectively. In comparison, the SEL at the axial end (tips) of the PEL (SELLA), where the most elongation was expected to occur, were almost 3 times longer in the positive-control group (P < 0.001).

**Fig 8:**
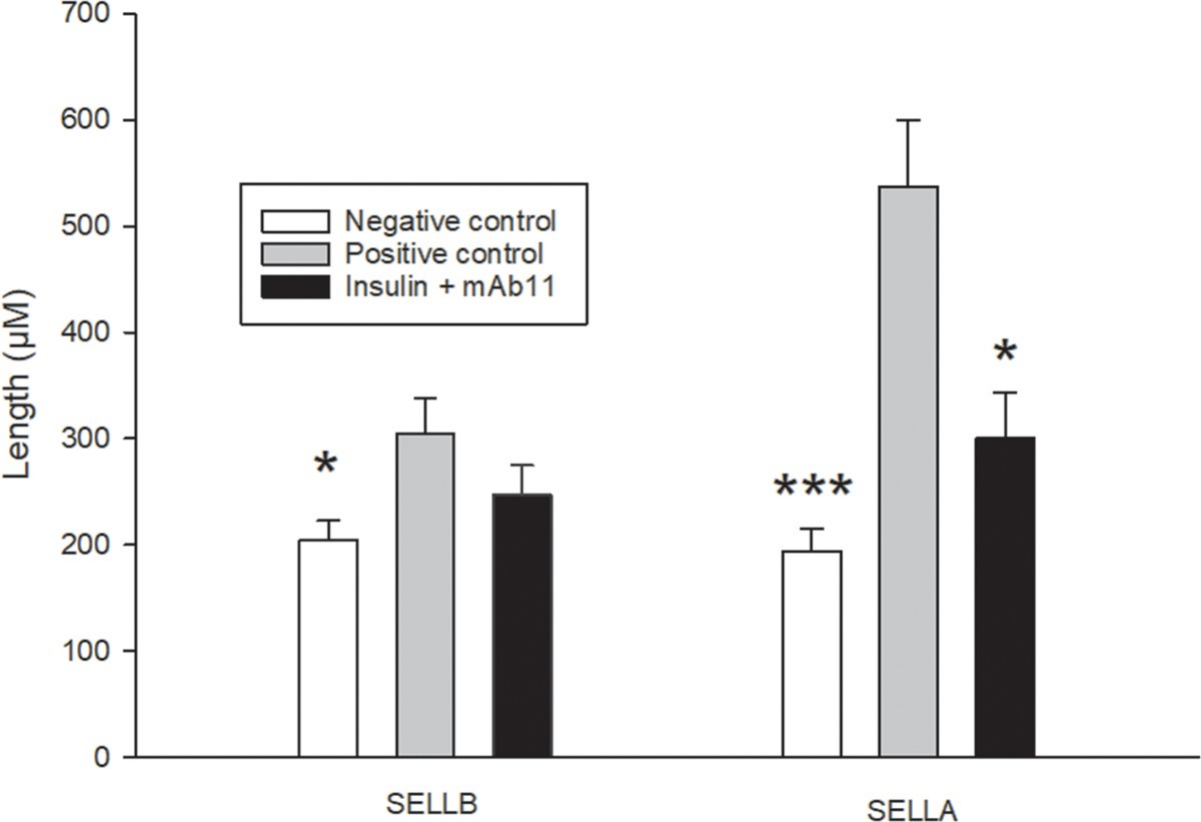
The comparative effects of insulin and mAb11 on the length of secondary epidermal lamellae at the base (abaxial, SELLB) and tips (axial, SELLA) of the primary epidermal lamellae in three groups of horses. Horses were treated with 0.9% saline (negative-control, n = 6); a combination of insulin and glucose infusion (positive-control, n = 6); or a combination of insulin and glucose, plus an anti-IGF-1R monoclonal antibody (mAb11, n = 7). Data are shown as mean + SE. *P < 0.05 and ***P < 0.001 indicate significant differences compared to the positive-control group.

**Fig 9:**
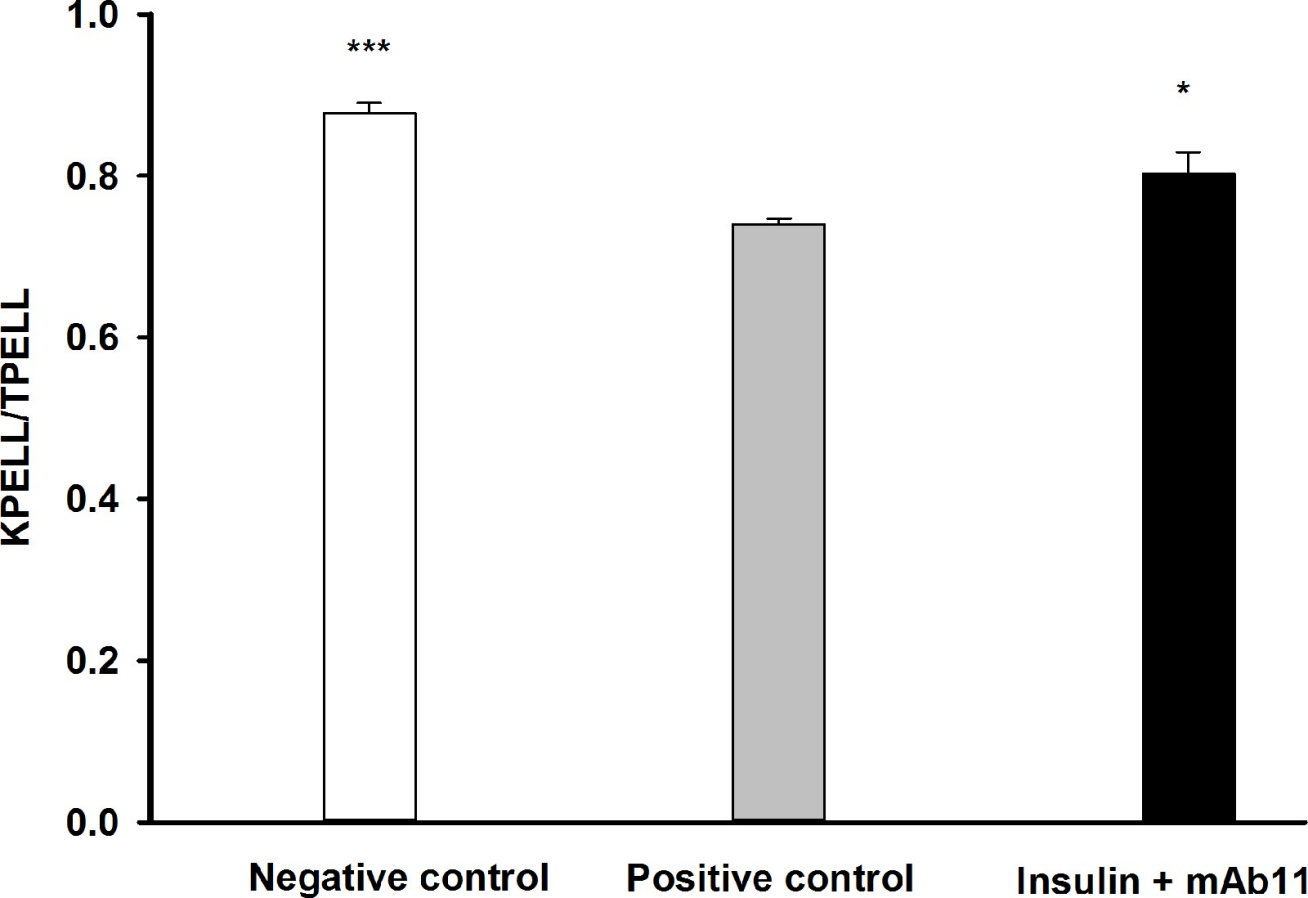
The ratio of keratinised to total primary epidermal lamellar length (KPELL/PELL) in 8 consecutive PEL from three groups of horses. The groups received: 0.9% saline (positive-control, n = 6), a combined insulin and glucose infusion (positive-control, n = 6), or a combination of insulin, glucose and an anti-IGF-1R monoclonal antibody (mAb11, n = 7). *P < 0.05 and ***P < 0.001 indicate significant differences compared to the positive-control group.

When the positive-control group was compared with the group that was pre-treated with mAb11, most of the variables measured were not significantly different. A clear exception, however, was in the region where the most damage occurred. At the axial end of the PEL, the SEL length (SELLA) was increased by 0.5-fold, rather than 1.8-fold (P < 0.05). The contrasting effects of treatment on the SEL at the abaxial (base) and axial end of the PEL, is illustrated in Fig 8. Similarly, there was a beneficial effect of mAb11 (P < 0.05) on another indication of damage to the tissue, which is the relative length of keratinised PEL to total PEL (KPELL: TPELL ratio; Fig 9).

## Discussion

One aim of this study, to induce laminitis in a group of healthy horses using a modified p-EHC technique, was clearly achieved. All thirteen horses that received insulin also developed laminitis, based on clinical observations, radiographic data and histological analysis. This result adds to a growing body of evidence indicating that hyperinsulinaemia is a critical and causal factor in laminitis [6].

Hyperinsulinemia has been confirmed by several scientists to be a primary predictor of laminitis risk [19, 20], and the induction of laminitis using a p-EHC has been demonstrated previously in normal ponies [5] and Standardbred horses [6]. However, previous studies using the p-EHC have been criticized and their clinical relevance questioned, on the basis that the insulin concentrations that were maintained, were beyond the physiological range seen in most insulin dysregulated horses. In the first study of this kind, Asplin *et al*. [5] maintained insulin concentrations of about 1,500 μIU/ml in ponies for up to 72 h, while those achieved by de Laat *et al*. [6] ranged from 1,000 to 1,200 μIU/ml over 48 h. In the present study, by reducing the dose infused, serum insulin concentrations ranged from only 300 to 500 μIU/ml, which is well within the range seen naturally in insulin-dysregulated ponies fed a high sugar diet [4]. Thus, although the infusion model causes sustained hyperinsulinemia, which may not mimic the natural situation precisely, demonstrating that laminitis can be induced in healthy horses at insulin concentrations that fall within the high physiological range, strongly supports the conclusion that hyperinsulinaemia is a critical factor in the disease.

This study also employed three useful and practical refinements of the original experimental model [5], while developing a procedure that is more humane, equally effective, and less expensive to perform, both in terms of materials and labour. First, the shorter experimental period used in this study and in the study by de Laat *et al* [6], reduces the time during which the animals must be subjected to confinement, by one third, which is consistent with the welfare principles of good practice in animal studies: the replacement, reduction, or refinement of techniques that employ animals in research [21]. Secondly, use of the ‘modified Obel’ method to assess lameness [11], is a further refinement which allowed some of the horses to receive pain relief before attaining a score of Obel grade 1 laminitis. This would also have helped to reduce their discomfort. Finally, in a previous study, radiographs showed no significant changes when they were taken before and after the experimental induction of laminitis in Standardbred horses through the infusion of insulin for 48 h [6]. However, significant changes were able to be detected after 48 h in the current study, by using permanent radiopaque reference markers on the dorsal hoof wall and sole, together with digital radiographic technology. As far as the authors are aware, this is the first time that laminitis pathology has been diagnosed radiographically at such an early stage, and shows the value of standardised digital technique, permanent radiopaque reference markers, and measuring not only the distance between outer hoof wall and distal phalanx, but also the distance between sole and the distal phalanx. These measurable changes support the notion that the clinical signs of laminitis foot pain are due to lamellar disintegration and the concommittant downward, painful, descent of the distal phalanx onto the dorsal solear dermis [22].

In terms of radiographic changes, it should be acknowledged that despite this improved technique, we were unable to accurately determine any change in founder distance, as observed by others when far more severe cases of laminitis have been studied over a much longer timeframe [23]. The mean founder distance in all feet before treatment was 2 mm, with a coefficient of variation (CV) of 93%. In a study using 36 healthy horses, others have reported an average founder distance of 4.6 mm, with a CV of 53% [24]. This contrasts with the HW to DP distances which averaged 18 to 19 mm, with a coefficient of variation of 7 to 9%, allowing these measurements to be made, and changes detected, with greater accuracy.

Another unexpected observation was that insulin concentrations were slightly elevated at time 0, relative to the values expected in young, healthy, insulin-sensitive Standardbred horses. In fact, before the infusions commenced, we observed that insulin concentrations were low (and within the healthy range) initially, but that they had increased by time 0. This may have been due to stress associated with the pre-infusion procedures, but was clearly abated, as insulin levels had fallen to their initial values in the control group at the time of the next blood sample, and remained low throughout the experiment.

A separate aim of the current study was to determine if mAb11 can prevent or attenuate the effects of hyperinsulinaemia, in terms of clinical signs, radiography and lamellar histopathology. Although the results do not support the hypothesis that mAb11 prevents laminitis (at the given dose), they do provide evidence that the antibody can lessen some of the effects of high insulin concentrations in Standardbred horses. Our conclusion in this regard is based primarily on a quantitative histological analysis of primary and secondary lamellae elongation, as well as radiographic analysis.

Nevertheless, the implications of the present findings need to be considered carefully, and caution is needed when drawing inferences from the results, both concerning the mechanism of insulin action, and the efficacy of the mAb11 treatment. It should be acknowledged that many variables were measured in this study, and that some findings are clearer than others. For example, even though some significant differences were observed in the left or right foot, we have also presented data for the combined feet, to reduce the variability and further interrogate specific data.

Although a qualitative analysis of the lamellar tissue by two blinded experts revealed no marked effects of the antibody, a quantitative histomorphometric analysis did reveal some important changes in response to both insulin and the mAb. It is important to note though, that the effects of insulin alone on the lamellar tissue were uneven. Whereas certain regions of the lamellae showed a modest response to insulin (e.g. a 48% increase in the length of the SEL at the abaxial end of the PEL), other regions showed a much larger response (i.e. a 1.8-fold increase in SEL length at the opposite, axial end of the PEL). These results are entirely consistent with earlier experiments [6], and in presenting these data we have chosen to focus on the region of the tissue where the response to insulin was greatest, believing that the effect of the mAb11 could be judged with most confidence here. It is possible that the lack of a statistically significant effect in other regions could reflect a failure of the antibody, but the authors believe it more likely that the change was too small to detect, and thus the experiment was underpowered with respect to these variables. For example, with the power of our analysis (ß) set to 0.8 and with α at 0.05, the effect of insulin on the SEL at the abaxial end of the PEL would need to have been reduced by the mAb by 90%, before the effect of the mAb reached statistical significance.

Balanced against some compelling data that mAb11attenuated the effects of insulin on certain histological variables, is the practical clinical observation that the treatment did not eliminate, attenuate or delay the signs of lameness. Demonstrating mAb11 is capable of doing so, will be critical to the future development of this treatment.

It was intended to treat only one foot with mAb11, in order to achieve a relatively high concentration of antibody locally, and a concomitant low concentration systemically, which was not expected to affect the other feet. However, there were two inherent risks with this approach. The first risk was that the antibody would either be too large, or not have sufficient time, to leave the circulation and penetrate the lamellar tissue, as the tourniquet could not be left in place for more than 20 min without chancing injury to the horse. This risk could have been avoided by administering mAb11 systemically, but insufficient material was available to do this while achieving the intended local concentration.

The second risk was that the antibody (and/or the local anaesthetic) might prevent or diminish pain in the treated foot, encouraging the horse to put more weight on that foot, and suffer more damage as a consequence. Indeed, an unexpected observation in the present study was that the damage caused by insulin was not even across both feet. This is difficult to explain, given that the insulin was administered systemically. Conversely, and equally unexpectedly, the ability of mAb11 to attenuate this damage did not appear to show a bias between feet, whereas this agent was delivered locally into one foot only. The biased effect of insulin alone could have been due to differences in load bearing, as discussed above, but given that the local anaesthetic has a short duration of action, there may be other explanations. For example, it is possible that these Standardbreds might have had some underlying damage to their left feet due to having run habitually in a counter-clockwise direction during their racing career. In terms of the mAb failing to show selectivity between feet, antibodies are normally transported across the vascular endothelium via an active uptake mechanism, so a possible explanation for this finding is that there was insufficient time for the antibody to effectively cross the lamellar capillary walls and the lamellar epidermal basement membrane during the 20 min that the tourniquet was in place. Therefore, much of the active transport must have occurred from the circulation after removal of the tourniquet (and distribution of the antibody in the systemic circulation). Because the antibody seems to have achieved an active concentration in the systemic circulation, we cannot rule out the possibility that it blunted the effects of IGF-1 and on other tissues and organs. In fact, several potential mAb therapies for cancer have failed to progress beyond phase III clinical trials, due to adverse effects such as hyperglycaemia and metabolic syndrome [25]. Having not anticipated such effects following a much shorter-term treatment in the horse, however, we have no observations to support or refute this possibility.

As the difference between the left and right feet was not consistent in all horses, and because the values for individual feet showed a large degree of variation, which could have masked real changes in response to treatment, the result for both feet were combined to reduce the variance and obtain a clearer picture of the insulin and antibody responses.

The dose of mAb11 used for this project (20 mg/per horse) was based on the amount of similar antibodies used to treat cancer in humans [14, 15], and adjusted for the estimated weight of one foot. Given that the average weight of the horses used in this study was 445 kg, this represents a dose of only 45 μg/kg BW systemically. Thus, it is possible that if the antibody was given systemically at a larger dose, approaching the human therapeutic dose, it may have been much more effective at competing with, or counteracting the effects of insulin, as we have seen in our studies *in vitro*, where the effects of insulin on cell proliferation were inhibited completely [13].

By partly, but not fully, counteracting some of the effects of insulin, the results with mAb11 raise important questions about the mechanism of insulin action. Because some antagonistic effects were seen using mAb11, which is highly-selective for blocking IGF-1R, it could be concluded that at least part of the laminitic effects of insulin are mediated via the activation of IGF-1R. This is consistent with the finding that insulin-stimulated cell proliferation in isolated lamellar cells can be blocked by a selective anti-IGF-1R mAb [10].

The inability of mAB11 to prevent laminitis could have been due to the fact that the dose of mAb11 was too low, or due to a parallel mechanism which may or may not involve insulin receptors. Furthermore, the ability of insulin to activate IGF-1R when it has such a low binding affinity for this receptor [7], is a mystery. Clearly, further work is needed to fully understand the mechanism of insulin action, and the availability of larger quantities of mAb11 will be helpful in this regard.

In conclusion, this study has provided further evidence that IGF-1R may play a role in the pathogenesis of endocrinopathic laminitis, and more investigations using higher doses of mAb11 are needed to determine how important this role is. Meanwhile, the information from this study will be useful in clarifying the direction of future research, and the modified p-EHC method described here could provide a useful tool in these endeavours.

## Supporting information

S1 Fig Lateral-medial radiographic image of a horse’s foot, illustrating the measurements made to detect the onset of laminitis. The distance between the outer hoof wall (HW) and the parietal cortex of the distal phalanx (DP) was measured at three points in the proximal (A), middle (B) and distal (C) regions. The distance from the tip of the DP to the sole of the foot (D) was also measured.
(DOCX)

S2 Fig Transverse sections of lamellar tissue from the middle lamellae of a foot from a negative control horse. Lines indicates the technique used for histomorphometry measurements, which included: the total length of each primary epidermal lamellar (TPELL); the length of the keratinised section of each primary epidermal lamellar (KPELL); the length of 10 consecutive secondary epidermal lamellar (SELL) in the abaxial (SELLB) and axial (SELLA) regions of all PELs, for each foot; the width of 10 secondary epidermal lamellar (SELW) in the upper-side mid-section, and 10 in the lower-side mid-section (with the basement membrane on the right), of all eight PELs for every section.
(DOCX)

S1 Table Histomorphometry measurements (mean ± SE; in μm) in forelimb hoof lamellar sections of three groups of horses treated with a balanced 0.9% saline (negative-control, n = 6), a combination of insulin and glucose (positive-control, n = 6), and an equine anti-IGF-1R monoclonal antibody (mAb11, n = 7). Eight primary epidermal lamellae (PEL) were measured, and total and keratinized lengths recorded. The length of 10 secondary epidermal lamellae (SEL) was measured at both the base and tip of all 8 PEL. The width of 10 SEL was measured in the mid-section of each PEL. No effect of section location on the measurements was observed.
(DOCX)

S2 Table Median (range) histology scores and mean (± SE) change (Δ) in the distance between the distal phalanx (DP) and the hoof wall (HW) at three points relative to the coronet, and the distal phalanx and sole of the foot, after a 48 h period of infusion with saline, insulin, or insulin plus an anti-IGF-1 receptor monoclonal antibody (mAb11). (DOCX)
