# Supplementary material for: Effects of an anti-IGF-1 receptor monoclonal antibody on laminitis induced by prolonged hyperinsulinaemia in Standardbred horses
Source: PLoS One. 2020 Sep 29;15(9):e0239261. doi: 10.1371/journal.pone.0239261 (PMC7524003; doi:10.1371/journal.pone.0239261)
Supplement: S1 Fig — The distance between the outer hoof wall (HW) and the parietal cortex of the distal phalanx (DP) was measured at three points in the proximal (A), middle (B) and distal (C) regions. The distance from the tip of the DP to the sole of the foot (D) was also measured. (DOCX) [file pone.0239261.s001.docx]

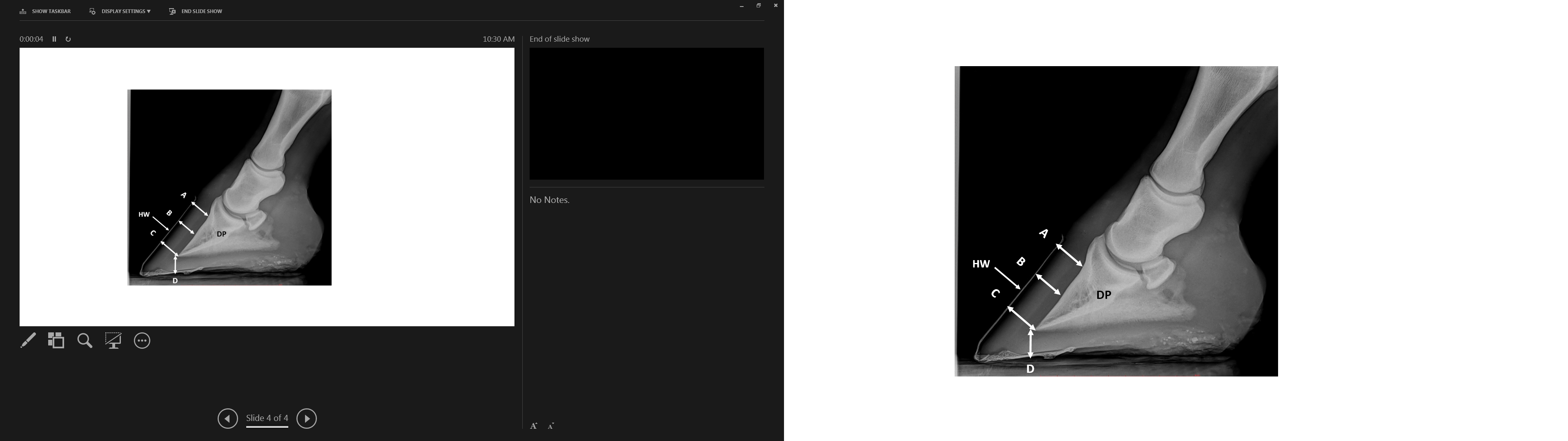


**S1 Fig. Lateromedial radiographic image of a horse’s foot, illustrating the measurements made to detect the onset of laminitis.** The distance between the outer of hoof wall (HW) and the parietal cortex of the distal phalanx (DP) was measured at three points in the proximal (A), middle (B) and distal (C) regions. The distance from the tip of the DP to the sole of the foot (D) was also measured.
