# Supplementary material for: Effects of an anti-IGF-1 receptor monoclonal antibody on laminitis induced by prolonged hyperinsulinaemia in Standardbred horses
Source: PLoS One. 2020 Sep 29;15(9):e0239261. doi: 10.1371/journal.pone.0239261 (PMC7524003; doi:10.1371/journal.pone.0239261)
Supplement: S2 Fig — Lines indicates the technique used for histomorphometry measurements, which included: the total length of each primary epidermal lamellar (TPELL); the length of the keratinised section of each primary epidermal lamellar (KPELL); the length of 10 consecutive secondary epidermal lamellar (SELL) in the abaxial (SELLB) and axial (SELLA) regions of all PELs, for each foot; the width of 10 secondary epidermal lamellar (SELW) in the upper-side mid-section, and 10 in the lower-side mid-section (with the basement membrane on the right), of all eight PELs for every section. (DOCX) [file pone.0239261.s002.docx]

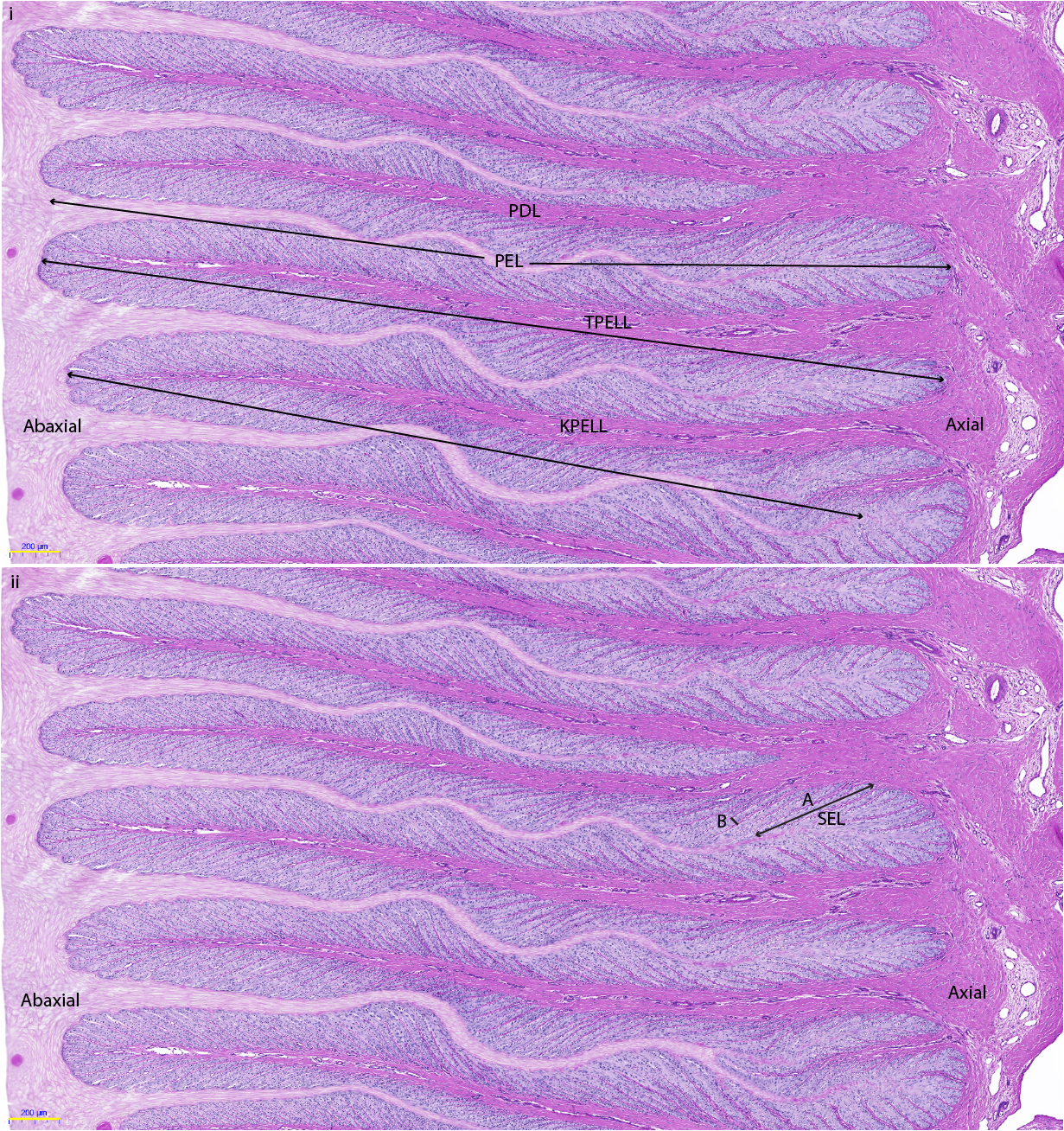


**S2 Fig.** **Transverse sections of lamellar tissue from the middle lamellae of a foot from a negative control horse.** Lines indicates the technique used for histomorphometry measurements, which included: the total length of each primary epidermal lamellar (TPELL); the length of the keratinised section of each primary epidermal lamellar (KPELL); the length of 10 consecutive secondary epidermal lamellar (SELL) in the abaxial (SELLB) and axial (SELLA) regions of all PELs, for each foot; the width of 10 secondary epidermal lamellar (SELW) in the upper-side mid-section (with the basement membrane/axial end oriented to the right, and 10 in the lower-side mid-section, of all eight PELs for every section.
