# Supplementary material for: Effects of an anti-IGF-1 receptor monoclonal antibody on laminitis induced by prolonged hyperinsulinaemia in Standardbred horses
Source: PLoS One. 2020 Sep 29;15(9):e0239261. doi: 10.1371/journal.pone.0239261 (PMC7524003; doi:10.1371/journal.pone.0239261)
Supplement: S1 Table — Eight primary epidermal lamellae (PEL) were measured, and total and keratinized lengths recorded. The length of 10 secondary epidermal lamellae (SEL) was measured at both the base and tip of all 8 PEL. The width of 10 SEL was measured in the mid-section of each PEL. No effect of section location on the measurements was observed. (DOCX) [file pone.0239261.s003.docx]

**S1 Table. Median (range) histology scores and mean (± SE) change (Δ) in the distance between the distal phalanx (DP) and the hoof wall (HW) at three points relative to the coronet, and the distal phalanx and sole of the foot, after a 48 h period of infusion with saline, insulin, or insulin plus an anti-IGF-1 receptor monoclonal antibody (mAb11).**

|  | **Left foot** | **Right foot** |
| --- | --- | --- |
| **Histology score, scale 1-4** |  |  |
| Negative control (saline) | 0 (0-1) | 0 (0-0.5) |
| Positive control (insulin) | 2.25 (1-3) | 2.5 (1-3) |
| Treatment (insulin + mAb11) | 1.50 (0.5-2.25) | 2 (1-3) |
|  |  |  |
| Δ Proximal HW-DP distance, mm |  |  |
| Negative control | -0.20 ± 0.52 | -0.36 ± 0.47 |
| Positive control (insulin) | 1.06 ± 0.32 | 0.14 ± 0.45 |
| Treatment (insulin + mAb11) | 0.32 ± 0.18 | -0.19 ± 0.25 |
|  |  |  |
| Δ Medial HW-DP distance, mm |  |  |
| Negative control | -0.08 ± 0.50 | 0.04 ± 0.38 |
| Positive control (insulin) | 1.18 ± 0.20 | 0.50 ± 0.55 |
| Treatment (insulin + mAb11) | 0.65 ± 0.20 | 0.31 ± 0.22 |
|  |  |  |
| Δ Distal HW-DP distance, mm |  |  |
| Negative control | -0.41 ± 0.60 | 0.26 ± 0.26 |
| Positive control (insulin) | 1.73 ± 0.38 | 1.08 ± 0.67 |
| Treatment (insulin + mAb11) | 0.99 ± 0.38 | 0.37 ± 0.13 |
|  |  |  |
| Δ DP to sole distance, mm |  |  |
| Negative control | 0.32 ± 0.28 | 0.49 ± 0.20 |
| Positive control (insulin) | -0.92 ± 0.16 | -0.91 ± 0.29 |
| Treatment (insulin + mAb11) | -0.58 ± 0.21 | -0.47 ± 0.20 |
