# Supplementary material for: Effects of an anti-IGF-1 receptor monoclonal antibody on laminitis induced by prolonged hyperinsulinaemia in Standardbred horses
Source: PLoS One. 2020 Sep 29;15(9):e0239261. doi: 10.1371/journal.pone.0239261 (PMC7524003; doi:10.1371/journal.pone.0239261)
Supplement: S2 Table — (DOCX) [file pone.0239261.s004.docx]

**S2 Table. Histomorphometry measurements (mean ± SE; in µm) in forelimb hoof lamellar sections of three groups of horses treated with a balanced electrolyte solution (negative-control, n=6), a combination of insulin and glucose (positive-control, n = 6), and an equine anti-IGF-1R monoclonal antibody (mAb11, n = 7). Eight PEL were measured, and total and keratinized lengths recorded. The length of 10 SEL was measured at both the base (abaxial end) and tip (axial end) of all 8 PEL. The width of 10 SEL was measured in the mid-section of each PEL. No effect of section location on the measurements was observed.**

| **Group** | | **TPELL** | **KPELL** | **SELLB** | **SELLA** | **SELWU** | **SELWL** |  |
| --- | --- | --- | --- | --- | --- | --- | --- | --- |
| *Negative-control* | | | | | | | | |
| Right | 3061 ±133 | | 2707 ± 140 | 181 ± 19 | 178 ± 20 | 23.3 ± 1.9 | 23.2 ± 2.2 |  |
| Left | 3253 ± 100 | | 2835 ± 105 | 230 ± 70 | 209 ± 32 | 23.5 ± 2.1 | 24.3 ± 2.1 |  |
| Both feet | 3157 ± 107^***^ | | 2771 ± 105^*^ | 205 ± 18^*^ | 194 ± 21^***^ | 23.4 ± 1.9^***^ | 23.8 ± 2^***^ |  |
| *Positive control (insulin)* | | | | | | | | |
| Right | 4069 ± 84 | | 2983 ± 92 | 293 ± 50 | 588 ± 100 | 12.5 ± 1.1 | 11.3 ± 1.2 |  |
| Left | 4093 ± 98 | | 3056 ± 112 | 313 ± 22 | 485 ± 45 | 12.0 ± 1.4 | 11.3 ± 1.2 |  |
| Both feet | 4081 ± 64 | | 3019 ± 60 | 304 ± 34 | 537 ± 63 | 12.3 ± 1.1 | 11.3 ± 1.1 |  |
| *Insulin + mAb11* | | | | | | | | |
| Right | 4140 ± 279 | | 3303 ± 224 | 243 ± 19 | 317 ± 41 | 13.4 ± 1.09 | 12.4 ± 0.99 |  |
| Left | 3719 ± 203 | | 2977 ± 157 | 251 ± 38 | 284 ± 48 | 15.0 ± 0.90 | 14.1 ± 0.82 |  |
| Both feet | 3939 ± 224**^*^** | | 3104 ± 173 | 247 ± 28 | 300 ± 43**^*^** | 14.2 ± 0.64 | 13.3 ± 0.48 |  |

TPELL: total primary epidermal lamellar length; KPELL: keratinized primary epidermal lamellar length; SELLB: length of the secondary epidermal lamellae at the abaxial end of the PEL; SELLA: length of the secondary epidermal lamellae at the axial end of the PEL; SELWU: secondary epidermal lamellar width at the midpoint of upper side of the PEL, SELWL: secondary epidermal lamellar width at the mid-point of the lower side of the PEL.

*P < 0.05 and ***P < 0.001 for comparison with the positive-control group.
